# Une métastase intra-thyroïdienne révélant un cancer bronchique non à petites cellules

**DOI:** 10.11604/pamj.2015.22.189.6536

**Published:** 2015-10-23

**Authors:** Anwar Boukir, Mustapha El Kabous, Ilham Azghari, Saber Boutayeb, Ibrahim El Ghissassi, Hind Mrabti, Hassan Errrihani

**Affiliations:** 1 Université Mohammed V, Faculté de Médecine et de Pharmacie, Institut National d’ Oncologie, Rabat, Maroc; 2 Université Mohammed V, Faculté de Médecine et de Pharmacie, CHU Avicenne, Rabat, Maroc

**Keywords:** Métastase intra-thyroïdienne, cancer bronchique, dysphonie, intra-thyroid metastasis, bronchial carcinoma, dysphonia

## Abstract

Les métastases thyroïdiennes sont très peu fréquentes. Elles peuvent de façon exceptionnelle révéler le cancer primitif. Nous rapportons le cas d'une patiente qui a présenté une dysphonie secondaire à un gros nodule thyroïdien lobaire droit. L'examen anatomopathologique de la pièce de l'hémi thyroïdectomie a révélé la présence d'un adénocarcinome d'origine pulmonaire. Le bilan d'extension a confirmé la présence d'une masse au niveau du Fowler droit ainsi qu'une métastase du trochanter fémoral droit et une récidive au niveau de la loge thyroïdienne. Une chimiothérapie à base de Paclitaxel, Carboplatine et Bevacizumab a été débuté. L’évaluation après 4 cures est en faveur d'une stabilité radiologique avec amélioration des symptômes.

## Introduction

Les métastases thyroïdiennes sont très peu fréquentes. Elles apparaissent le plus souvent au cours du suivi d'un patient traité mais peuvent être de façon exceptionnelle révélatrice du cancer primitif. Ce dernier cas de figure a été rarement rapporté dans la littérature.

## Patient et observation

**Figure 1:**
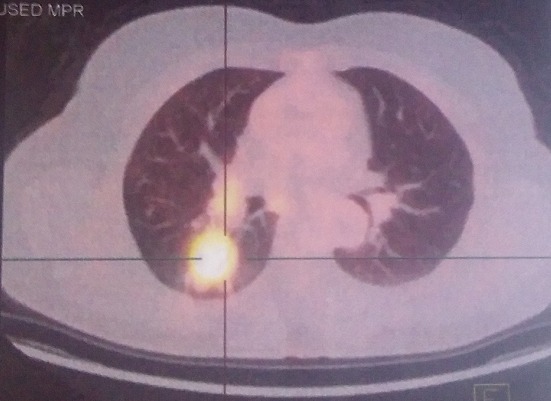
La masse pulmonaire droite sur un foyer hyper métabolique (SUV max 7,54; Volume métabolique 17,68 cm3) avec un hyper métabolisme hilaire droit (SUV max 5,56) et une adénopathie hyper métabolique sous carinaire (SUV max 4,35)

**Figure 2:**
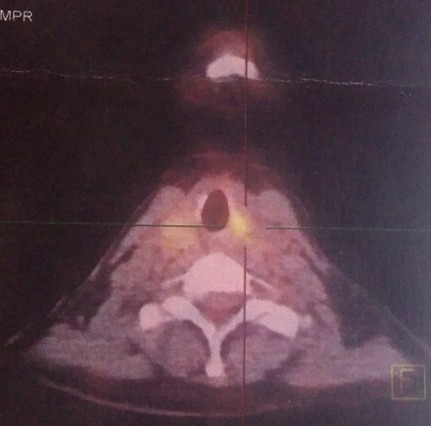
Foyer hyper métabolique de la loge thyroïdienne postérieur gauche (SUV max 3,59) compatible avec une récidive ou une maladie néoplasique résiduelle

Mme G.B âgée de 51 ans, une femme nord-africaine, sans antécédents particuliers et qui a présenté une dysphonie concomitante à l'apparition d'une masse cervicale droite. L'examen clinique initial trouve une tuméfaction dure, bien limitée et légèrement douloureuse au niveau du lobe gauche de la thyroïde. Le reste de l'examen clinique est sans particularités. il n'y'avait pas de signes cliniques ou biologiques de dysthyroïde. Une échotomographie thyroïdienne a montré la présence d'un nodule occupant les deux tiers inférieurs du lobe gauche très hypo échogéne, hétérogène, bien limité, hyper vascularisé mesurant 18\21\27 mm, classé TIRADS 4b. Une hémi thyroïdectomie gauche a été réalisée. L'examen anatomopathologique de la pièce opératoire trouve un adénocarcinome moyennement différencié. L'immuno histochimie est en faveur d'une origine pulmonaire avec des anticorps anti CK7 positifs, anticorps anti CK20 négatifs, anticorps anti TTF1 positifs, anticorps anti NAPSIN A positifs et anticorps anti thyroglobuline négatifs. Le scanner cervico-thoraco-abdomino-pelvien montre l'existence d'une masse de densité tissulaire et à contours irréguliers au niveau du Fowler droit mesurant 40\50 mm ainsi qu'une adénopathie hilaire homolatérale. La tomographie par émission de positron confirme la présence de la masse pulmonaire droite sur un foyer hyper métabolique (SUV max 7,54; Volume métabolique 17,68 cm3) (Figure 1). Le TEP scanner a également permis d'objectiver la présence d'un hyper métabolisme hilaire droit (SUV max 5,56) et d'une adénopathie hyper métabolique sous carinaire (SUV max 4,35) ainsi qu'un foyer hyper métabolique du trochanter fémoral droit (SUV max 4,33) (Figure 1). Au niveau cervical le TEP scanner montre un foyer hyper métabolique de la loge thyroïdienne postérieur gauche (SUV max 3,59) compatible avec une récidive ou une maladie néoplasique résiduelle (Figure 2). Devant ce bilan d'extension positif confirmant la dissémination métastatique de la maladie, une chimiothérapie à base de Paclitaxel, Carboplatine et Bevacizumab est débutée. L’évaluation après 4 cures est en faveur d'une stabilité radiologique avec amélioration des symptômes.

## Discussion

Les métastases thyroïdiennes représentent environ 4% de la pathologie néoplasique thyroïdienne. leurs taux d'incidence est plus important dans les séries autopsiques [1]. Elles peuvent être synchrones ou métachrones de la maladie primitive et peuvent dans de rares cas révéler le cancer primitif [2]. La symptomatologie clinique n'est pas spécifique, l'interrogatoire revêt une importance capitale à la recherche d'antécédents néoplasiques. L’échographie n'est pas spécifique. Elle peut retrouver des lésions hypoéchogènes localisées, parfois calcifiées, plus ou moins suspectes, ou un aspect hyperéchogène en rapport avec un remaniement inflammatoire ou nécrotique. Le scanner peut visualiser des lésions hypodenses, souvent hétérogènes, avec un rehaussement modéré après injection du produit de contraste. Le TEP-scanner ne constitue pas un examen de choix pour explorer les lésions thyroïdiennes mais peut s'avérer utile dans la recherche de la tumeur primitive et dans le bilan d'extension [3]. Le bilan biologique thyroïdien est le plus souvent normal, néanmoins de rares cas d'hyperthyroïdie ont été rapportés [1]. Un examen morphologique et une analyse immuno histochimique sur la biopsie ou la pièce opératoire sont nécessaires et peuvent fortement orienter vers la tumeur primitive [4]. En dehors des indications palliatives de la chirurgie, la prise en charge curative se fait en fonction du type histologique et de la réséabilité de la tumeur primitive [1]. Le traitement médical par chimiothérapie est proposé en complément ou en l'absence de possibilités chirurgicales et se fait en tenant compte du type histologique de la tumeur primitive et de l’état général du patient.

## Conclusion

Malgré leur rareté, le diagnostic d'une métastase thyroïdienne doit toujours être évoqué devant une tuméfaction de la glande surtout en présence d'antécédents de cancer. Cette investigation doit être complétée par une cytoponction et par la réalisation d'un bilan d'extension adéquat.
